# Correction: Methylglyoxal, a glycolysis side-product, induces Hsp90 glycation and YAP-mediated tumor growth and metastasis

**DOI:** 10.7554/eLife.96613

**Published:** 2024-02-06

**Authors:** Marie-Julie Nokin, Florence Durieux, Paul Peixoto, Barbara Chiavarina, Olivier Peulen, Arnaud Blomme, Andrei Turtoi, Brunella Costanza, Nicolas Smargiasso, Dominique Baiwir, Jean L Scheijen, Casper G Schalkwijk, Justine Leenders, Pascal De Tullio, Elettra Bianchi, Marc Thiry, Koji Uchida, David A Spiegel, James R Cochrane, Craig A Hutton, Edwin De Pauw, Philippe Delvenne, Dominique Belpomme, Vincent Castronovo, Akeila Bellahcène

**Keywords:** Chicken, Human, Mouse

Nokin M-J, Durieux F, Peixoto P, Chiavarina B, Peulen O, Blomme A, Turtoi A, Costanza B, Smargiasso N, Baiwir D, Scheijen JL, Schalkwijk CG, Leenders J, De Tullio P, Bianchi E, Thiry M, Uchida K, Spiegel DA, Cochrane JR, Hutton CA, De Pauw E, Delvenne P, Belpomme D, Castronovo V, Bellahcène A. 2016. Methylglyoxal, a glycolysis side-product, induces Hsp90 glycation and YAP-mediated tumor growth and metastasis. *eLife* **5**:e19375. doi: 10.7554/eLife.19375.

Published 19 October 2016

It has been brought to our attention that in Figure 2—figure supplement 1 panel A is the same as panel D. This unfortunate duplication probably occurred at the step of sending high resolution figures to the publication staff.

The corrected panels A and D of Figure 2—figure supplement 1 are shown here:

**Figure 1:**
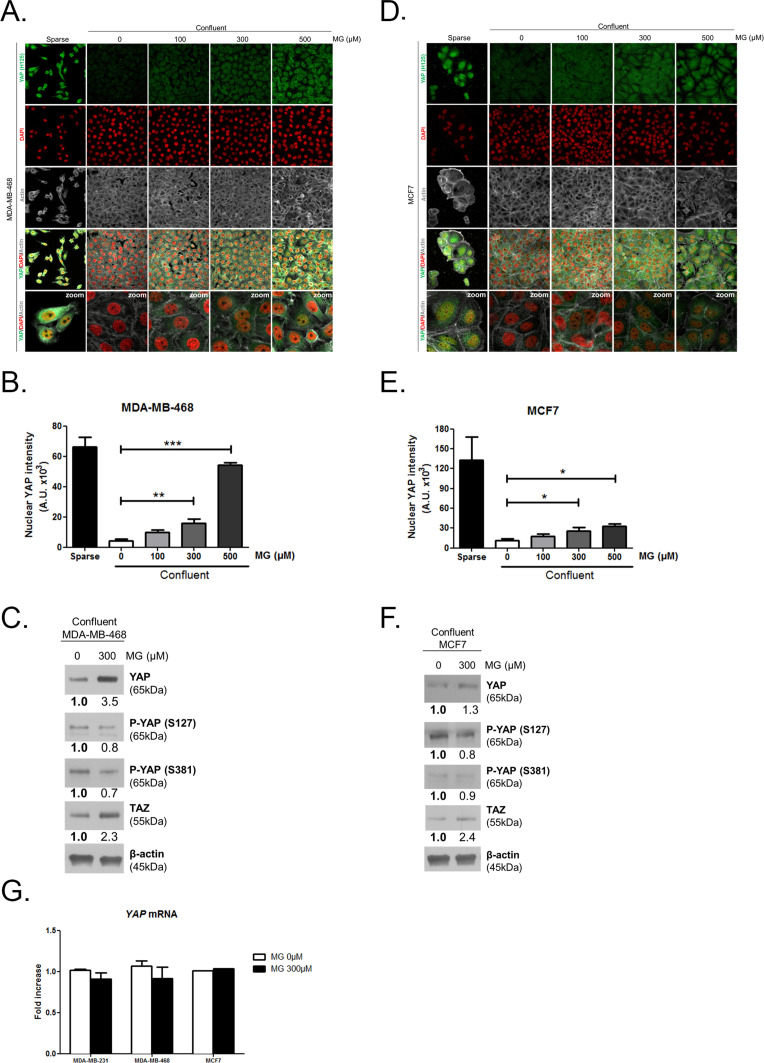


The originally published panels A and D of Figure 2—figure supplement 1 are shown for reference:

**Figure 2:**
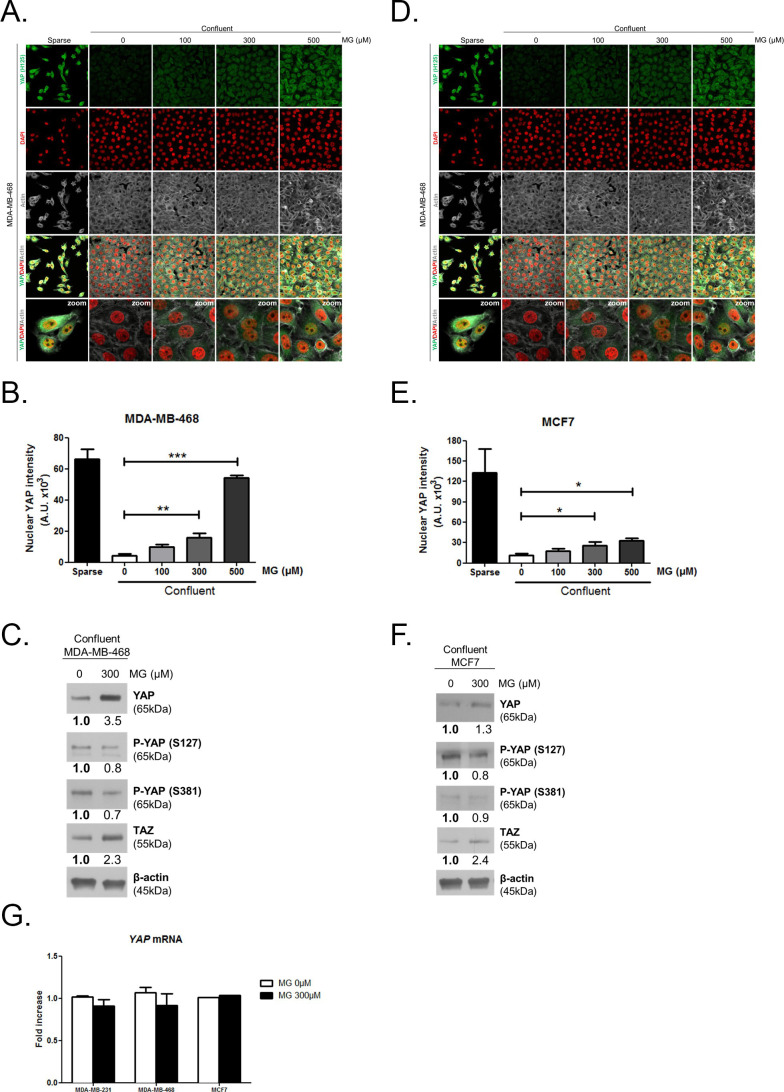


The article has been corrected accordingly.

