# Correction: Methylglyoxal, a glycolysis side-product, induces Hsp90 glycation and YAP-mediated tumor growth and metastasis

**DOI:** 10.7554/eLife.103327

**Published:** 2024-09-23

**Authors:** Marie-Julie Nokin, Florence Durieux, Paul Peixoto, Barbara Chiavarina, Olivier Peulen, Arnaud Blomme, Andrei Turtoi, Brunella Costanza, Nicolas Smargiasso, Dominique Baiwir, Jean L Scheijen, Casper G Schalkwijk, Justine Leenders, Pascal De Tullio, Elettra Bianchi, Marc Thiry, Koji Uchida, David A Spiegel, James R Cochrane, Craig A Hutton, Edwin De Pauw, Philippe Delvenne, Dominique Belpomme, Vincent Castronovo, Akeila Bellahcène

**Keywords:** Chicken, Human, Mouse

Nokin M-J, Durieux F, Peixoto P, Chiavarina B, Peulen O, Blomme A, Turtoi A, Costanza B, Smargiasso N, Baiwir D, Scheijen JL, Schalkwijk CG, Leenders J, De Tullio P, Bianchi E, Thiry M, Uchida K, Spiegel DA, Cochrane JR, Hutton CA, De Pauw E, Delvenne P, Belpomme D, Castronovo V, Bellahcène A. 2016. Methylglyoxal, a glycolysis side-product, induces Hsp90 glycation and YAP-mediated tumor growth and metastasis. *eLife* **5**:e19375. doi: 10.7554/eLife.19375.

Published 19 October 2016

We were notified via PubPeer of an error in Figure 2. In panel D, the immunofluorescence images corresponding to YAP detection in MDA-MB-231 cells cultured in presence of carnosine were duplicated resulting in identical images presented for YAP detection in MDA-MB-231 cells cultured under low glucose condition in Figure 3 panel F.

The inclusion of identical images in these figures was an unfortunate mistake. We suspect that this duplication occurred while selecting images from the dedicated folders where MDA-MB-231 IF raw data were classified. Going back to these files, we confirmed that images in Figure 2D were well attributed. Consequently, there are no changes to panel 2D.

We have since retrieved the images corresponding to YAP detection in MDA-MB-231 cells cultured under low glucose condition. These images have replaced the duplicated images in Figure 3 panel F.

Corrected Figure 3 is shown below:

**Figure 1:**
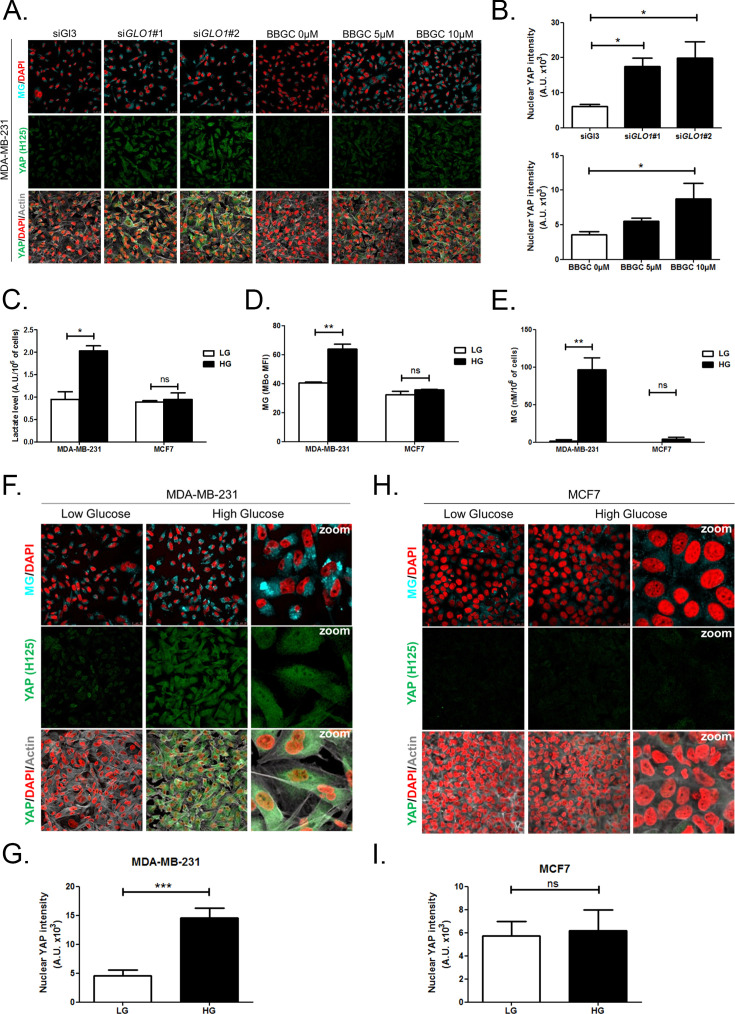


Originally published Figure 3 is shown here for reference:

**Figure 2:**
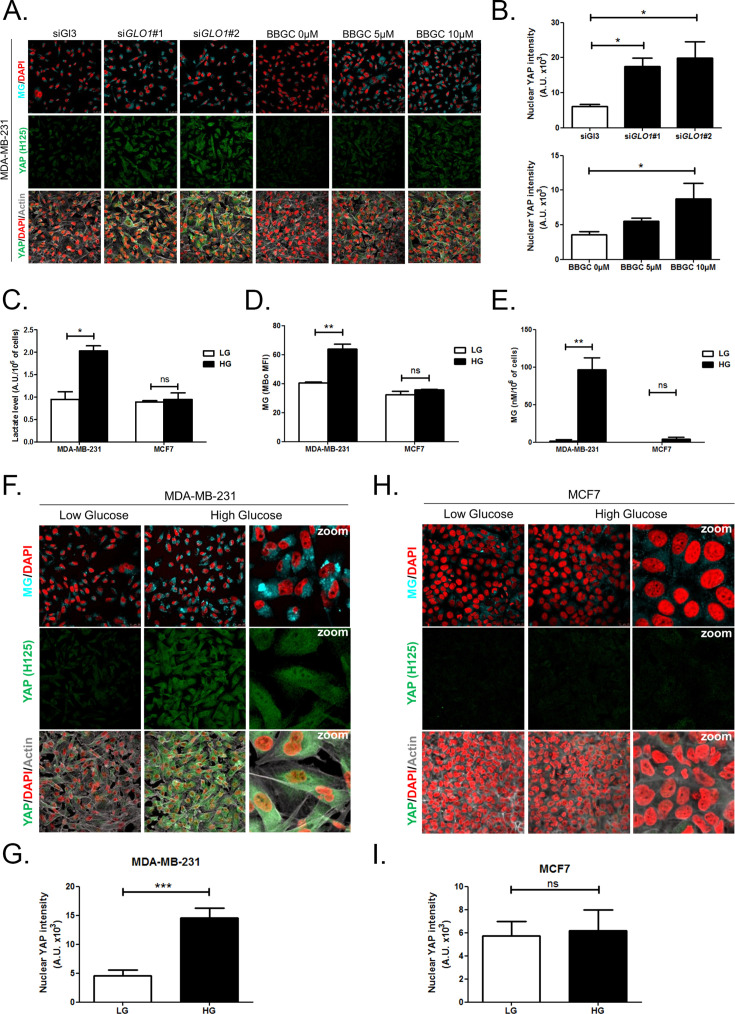


Upon the meticulous reviewing of all the picture panels, we observed an inaccuracy in Figure 2 panel A where the zoom figures were not appropriately addressed. We have now added boxes to the images to delimitate the zoomed regions in the corrected panel 2 A.

Corrected Figure 2 is shown below:

**Figure 3:**
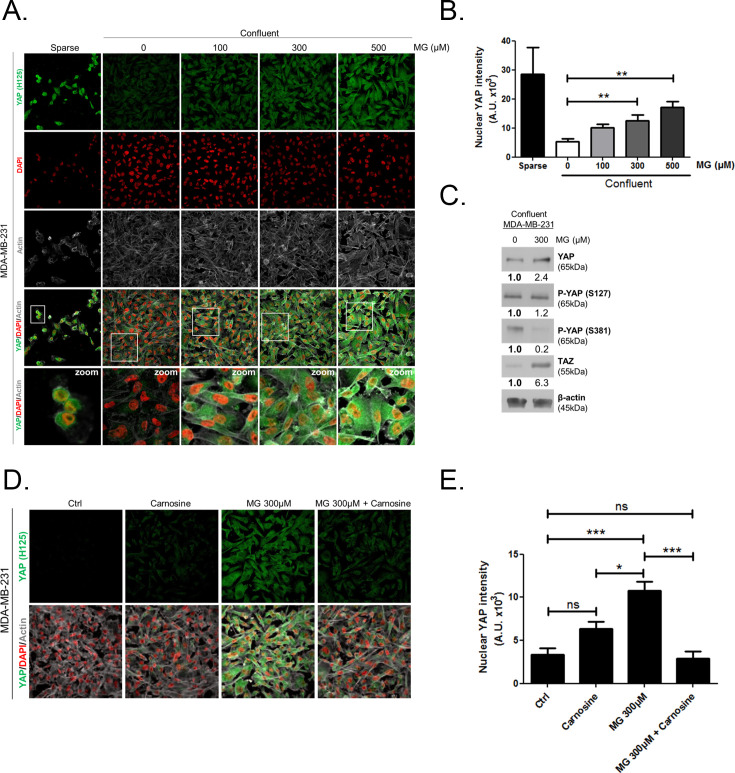


Originally published Figure 2 is shown here for reference:

**Figure 4:**
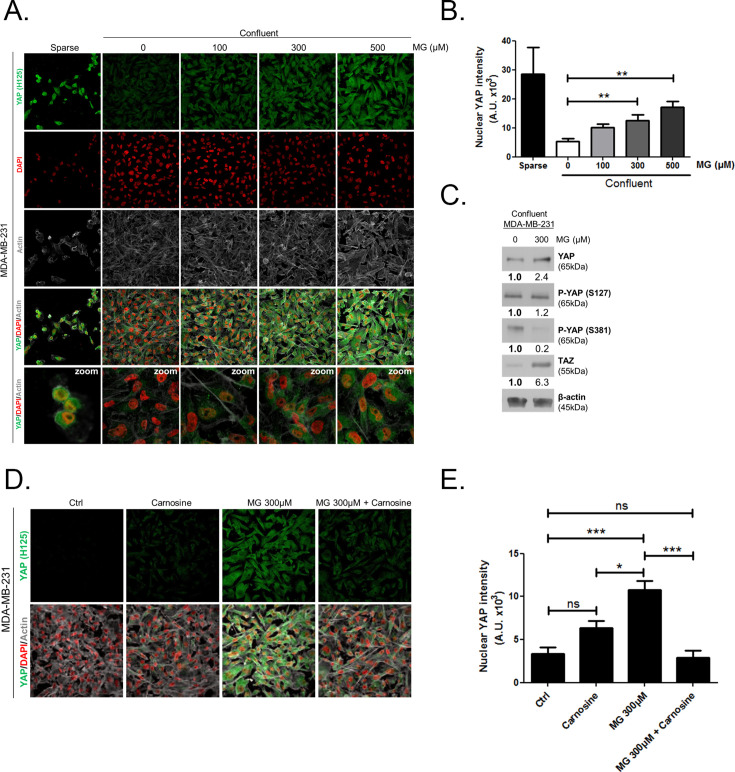


This article has already been corrected previously; please see https://doi.org/10.7554/eLife.96613 for further details. All the corrections do not affect the results and conclusions of the original article.

