# Supplementary material for: Methylglyoxal, a glycolysis side-product, induces Hsp90 glycation and YAP-mediated tumor growth and metastasis
Source: eLife. 2016 Oct 19;5:e19375. doi: 10.7554/eLife.19375 (PMC5081250; doi:10.7554/eLife.19375)
Supplement: Figure 7—source data 1. — Peptides identified by mass spectrometry of MG-Hsp90 enzymatic digests. Modification sites are bolded and underlined. CEL: Carboxyethyllysine. DOI: http://dx.doi.org/10.7554/eLife.19375.021 [file elife-19375-fig7-data1.docx]

**Figure 7 – source data 1. MG modifications on human recombinant Hsp90.** Peptides identified by mass spectrometry of MG-Hsp90 enzymatic digests. Modification sites are bolded and underlined. CEL: Carboxyethyllysine.

| **Start** | **End** | **Peptide sequence** | **Modifications** |
| --- | --- | --- | --- |
| 38 | 47 | YSNKEIFL**R**E | Dihydroxyimidazolidine |
| 47 | 61 | ELISNSSDALD**K**I**R**Y | Hydroimidazolone, Dihydroxyimidazolidine, Argpyrimidine, CEL |
| 48 | 61 | LISNSSDALDKI**R**Y | Hydroimidazolone, Dihydroxyimidazolidine |
| 49 | 61 | ISNSSDALDKI**R**Y | Dihydroxyimidazolidine |
| 52 | 61 | SSDALDKI**R**Y | Hydroimidazolone |
| 59 | 76 | I**R**YESLTDPSKLDSGKEL | Dihydroxyimidazolidine |
| 63 | 76 | ESLTDPSKLDSG**K**EL | CEL |
| 71 | 89 | DSGKELHINLIPNKQD**R**TL | Dihydroxyimidazolidine |
| 75 | 89 | ELHINLIPNKQD**R**TL | Hydroimidazolone |
| 76 | 89 | LHINLIPN**K**QD**R**TL | Dihydroxyimidazolidine, CEL |
| 78 | 89 | INLIPNKQD**R**TL | Dihydroxyimidazolidine |
| 84 | 98 | **K**QDRTLTIVDTGIGM | CEL |
| 84 | 100 | KQD**R**TLTIVDTGIGMTK | Dihydroxyimidazolidine |
| 99 | 112 | T**K**ADLINNLGTIAK | CEL |
| 101 | 116 | ADLINNLGTIA**K**SGTK | CEL |
| 143 | 153 | LVAE**K**VTVITK | CEL |
| 163 | 182 | ESSAGGSFTV**R**TDTGEPMGR | Dihydroxyimidazolidine |
| 171 | 182 | TV**R**TDTGEPMGR | Hydroimidazolone |
| 186 | 202 | VILHLKEDQTEYLEE**R**R | Hydroimidazolone, Dihydroxyimidazolidine |
| 189 | 202 | HLKEDQTEYLEE**R**R | Dihydroxyimidazolidine |
| 280 | 292 | IKE**K**YIDQEELNK | CEL |
| 298 | 313 | T**R**NPDDITNEEYGEFY | Dihydroxyimidazolidine |
| 314 | 329 | **K**SLTNDWEDHLAV**K**HF | CEL |
| 330 | 340 | SVEGQLEF**R**AL | Dihydroxyimidazolidine |
| 338 | 345 | **R**ALLFVPR | Dihydroxyimidazolidine |
| 346 | 352 | **R**APFDLF | Hydroimidazolone |
| 367 | 380 | **R**VFIMDNCEELIPE | Dihydroxyimidazolidine |
| 383 | 396 | NFI**R**GVVDSEDLPL | Dihydroxyimidazolidine |
| 385 | 401 | IRGVVDSEDIPLNLS**R**E | Dihydroxyimidazolidine |
| 459 | 466 | LSELL**R**YY | Hydroimidazolone, Dihydroxyimidazolidine |
| 465 | 483 | YYTSASGDEMVSL**K**DYCTR | CEL |
| 467 | 489 | TSASGDEMVSL**K**DYCT**R**MKENQK | Hydroimidazolone, Dihydroxyimidazolidine, CEL |
| 474 | 483 | MVSL**K**DYCTR | CEL |
| 493 | 511 | YITGET**K**DQVANSAFVE**R**L | CEL, Dihydroxyimidazolidine |
| 500 | 512 | DQVANSAFVE**R**LR | Argpyrimidine, Hydroimidazolone, Dihydroxyimidazolidine |
| 511 | 520 | LR**K**HGLEVIY | CEL |
| 529 | 541 | CVQQL**K**EFEG**K**TL | CEL |
| 535 | 546 | EFEG**K**TLVSVTK | CEL |
| 538 | 564 | G**K**TLVSVT**K**EGLELPEDEEEKKKQEEK | CEL |
| 586 | 597 | VVVSN**R**LVTSPC | Dihydroxyimidazolidine |
| 605 | 614 | GWTANME**R**IM | Hydroimidazolone, Dihydroxyimidazolidine |
| 613 | 627 | IMKAQAL**R**DNSTMGY | Dihydroxyimidazolidine |
| 616 | 628 | AQAL**R**DNSTMGYM | Dihydroxyimidazolidine |
| 632 | 647 | **K**HLEINPDHSIIETLR | CEL |
| 673 | 689 | SSGFSLEDPQTHAN**R**IY | Dihydroxyimidazolidine |
| 677 | 689 | SLEDPQTHAN**R**IY | Argpyrimidine, Dihydroxyimidazolidine |
| 694 | 732 | LGLGIDEDDPTADDTSAAVTEEMPPLEGDDDTS**R**MEEVD | Dihydroxyimidazolidine |
| 715 | 732 | EMPPLEGDDDTS**R**MEEVD | Hydroimidazolone, Dihydroxyimidazolidine |
