# Supplementary material for: Methylglyoxal, a glycolysis side-product, induces Hsp90 glycation and YAP-mediated tumor growth and metastasis
Source: eLife. 2016 Oct 19;5:e19375. doi: 10.7554/eLife.19375 (PMC5081250; doi:10.7554/eLife.19375)
Supplement: Figure 7—source data 2. — Peptides identified by mass spectrometry of argpyrimidine immunoprecipitate from MDA-MB-231 treated with MG (300 µM) during 6 hr. Modification sites are bolded and underlined.- DOI: http://dx.doi.org/10.7554/eLife.19375.022 [file elife-19375-fig7-data2.docx]

**Figure 7 – source data 2. MG modifications on endogenous Hsp90.** Peptides identified by mass spectrometry of argpyrimidine immunoprecipitate from MDA-MB-231 treated with MG (300µM) during 6h. Modification sites are bolded and underlined.

| **Start** | **End** | **Peptide sequence** | **Modifications** |
| --- | --- | --- | --- |
| 42 | 58 | EIFL**R**ELISNSSDALDK | Hydroimidazolone, Dihydroxyimidazolidine |
| 59 | 69 | I**R**YESLTDPSK | Hydroimidazolone, Dihydroxyimidazolidine |
| 85 | 100 | QD**R**TLTIVDTGIGMTK | Hydroimidazolone, Dihydroxyimidazolidine |
| 295 | 314 | PIWT**R**NPDDITNEEYGEFYK | Hydroimidazolone, Dihydroxyimidazolidine |
| 500 | 513 | DQVANSAFVE**R**L**R**K | Hydroimidazolone |
| 633 | 649 | HLEINPDHSIIETL**R**QK | Hydroimidazolone, Dihydroxyimidazolidine |
