# Supplementary material for: Methylglyoxal, a glycolysis side-product, induces Hsp90 glycation and YAP-mediated tumor growth and metastasis
Source: eLife. 2016 Oct 19;5:e19375. doi: 10.7554/eLife.19375 (PMC5081250; doi:10.7554/eLife.19375)
Supplement: Supplementary file 1. — DOI: http://dx.doi.org/10.7554/eLife.19375.027 [file elife-19375-supp1.docx]

**Supplementary file 1. Antibodies and dilution used for Western Blot experiments.** Research Resource Identifiers (RRIDs) from <https://scicrunch.org/resources> are mentioned, n.a: non available.

| **Protein targeted** | **Source** | **RRID** | **Clone/Cat#** | **Dilution (WB)** |
| --- | --- | --- | --- | --- |
| **Argpyrimidine** | Oya et al. JBC 1999 | n.a | mAb6B | 1/6000 |
| **β-actin** | Sigma-Aldrich (St Louis, MO, USA) | RRID:AB_476744 | A5441 | 1/5000 |
| **E-cadherin** | BD Biosciences (Franklin Lakes, NJ, USA) | RRID:AB_397580 | 610181 | 1/1000 |
| **Flag** | Sigma-Aldrich (St Louis, MO, USA) | RRID:AB_259529 | F3165 | 1/1000 |
| **Glyoxalase 1** | BioMAC (Leipzig, Germany) | n.a | #02-14 | 1/1000 |
| **Hsp27** | Cell Signaling (Danvers, MA, USA) | RRID:AB_331761 | #2402 | 1/1000 |
| **Hsp90** | Cell Signaling (Danvers, MA, USA) | RRID:AB_2233307 | #4877 | 1/1000 |
| **LATS1** | Bethyl (Montgomery, TX, USA) | RRID:AB_451012 | A300-477A | 1/1000 |
| **LATS2** | Cell Signaling (Danvers, MA, USA) | RRID:AB_10835233 | #5888 | 1/1000 |
| **MG-H (3D11)** | Cell Biolabs (San Diego, CA, USA) | n.a | STA-011 | 1/2000 |
| **Mst1** | Cell Signaling (Danvers, MA, USA) | RRID:AB_2144632 | #3682 | 1/1000 |
| **Mst2** | Cell Signaling (Danvers, MA, USA) | RRID:AB_2196471 | #3952 | 1/1000 |
| **Phospho-Smad2 (Ser465/467)/Smad3 (Ser423/425)** | Cell Signaling (Danvers, MA, USA) | n.a | #8828 | 1/500 |
| **P-YAP (S127)** | Cell Signaling (Danvers, MA, USA) | n.a | #13008 | 1/1000 |
| **P-YAP (S381)** | Cell Signaling (Danvers, MA, USA) | n.a | #13619 | 1/1000 |
| **SMAD2/3** | Cell Signaling (Danvers, MA, USA) | RRID:AB_10889933 | #8685 | 1/1000 |
| **TAZ** | BD Biosciences (Franklin Lakes, NJ, USA) | RRID:AB_1645338 | 560235 | 1/1000 |
| **Vimentin** | Sigma-Aldrich (St Louis, MO, USA) | RRID:AB_609914 | V6389 | 1/1000 |
| **YAP** | Santa Cruz (Santa Cruz, CA, USA) | RRID:AB_2273277 | sc-15407 | 1/1000 |
