# Supplementary material for: Methylglyoxal, a glycolysis side-product, induces Hsp90 glycation and YAP-mediated tumor growth and metastasis
Source: eLife. 2016 Oct 19;5:e19375. doi: 10.7554/eLife.19375 (PMC5081250; doi:10.7554/eLife.19375)
Supplement: Supplementary file 2. — DOI: http://dx.doi.org/10.7554/eLife.19375.028 [file elife-19375-supp2.docx]

**Supplementary file 2. siRNA sequences.**

| **Name** | **Sequence** |
| --- | --- |
| **siYAP#1** | 5’-AAAUAAAGCCAUUUCUGGUUUGCUCCU-3’ |
|  | 5’-AGGAGCAAACCAGAAAUGGCUUUAUUU-3’ |
| **siYAP#2** | 5’-ACUGGCAAAUUAUAGGCACUCCUUCCA-3’ |
|  | 5’-UGGAAGGAGUGCCUAUAAUUUGCCAGU-3’ |
| **siGlo1#1** | 5’-CUUGGCUUAUGAGGAUAAA-3’ |
|  | 5’-UUUAUCCUCAUAAGCCAAG-3’ |
| **siGlo1#2** | 5’-GAUGGCUACUGGAUUGAAA-3’ |
|  | 5’-UUUCAAUCCAGUAGCCAUC-3’ |
| **siGl3** | 5’-CUUACGCUGAGUACUUCGA-3’ |
|  | 5’-UCGAAGUACUCAGCGUAAG-3’ |
