# Supplementary material for: Methylglyoxal, a glycolysis side-product, induces Hsp90 glycation and YAP-mediated tumor growth and metastasis
Source: eLife. 2016 Oct 19;5:e19375. doi: 10.7554/eLife.19375 (PMC5081250; doi:10.7554/eLife.19375)
Supplement: Supplementary file 3. — DOI: http://dx.doi.org/10.7554/eLife.19375.029 [file elife-19375-supp3.docx]

**Supplementary file 3. Primer sequences and probes used for quantitative reverse transcription-PCR (qRT-PCR).**

| **Name** | **Fw/Rv** | **Sequence** | **Probe (UPL, Roche)** |
| --- | --- | --- | --- |
| **CTGF** | Fw | 5’-CCTGCAGGCTAGAGAAGCAG-3’ | 85 |
|  | Rv | 5’-TGGAGATTTTGGGAGTACGG-3’ |  |
| **YAP** | Fw | 5’-ATCCCAGCACAGCAAATTCT-3’ | 47 |
|  | Rv | 5’-TGGATTTTGAGTCCCACCAT-3’ |  |
| **LATS1** | Fw | 5’-GGCACAAACACCATTAGAAACA-3’ | 31 |
|  | Rv | 5’-AGAAGCTTCAGGACTGAGTTTAGC-3’ |  |
| **Hsp90** | Fw | 5’-GTCCTGTGCGGTCACTTAGC-3’ | 25 |
|  | Rv | 5’-AAAGGCGAACGTCTCAACC-3’ |  |
| **ANKFN1** | Fw | 5’-CCAGTGTGTTGAGGTGCATT-3’ | 64 |
|  | Rv | 5’-GCCCGAGAAAGTCCACACT-3’ |  |
| **RIMS3** | Fw | 5’-GTCTCCCCAGACGATCACC-3’ | 37 |
|  | Rv | 5’-AGCAGGCTCTGCTCTTTGAC-3’ |  |
| **KCNK1** | Fw | 5’-CTGACAAGGGCACGGTGT-3’ | 22 |
|  | Rv | 5’-GTCCTTCTTCGGCAGCAC-3’ |  |
| **EMP2** | Fw | 5’-CCGCAATCATGACACACAG-3’ | 42 |
|  | Rv | 5’-GAAGCAGGGAGAGAGGTTTG-3’ |  |
| **OSBP2** | Fw | 5’-CGTCGTCCCCAGAGTCAT-3’ | 1 |
|  | Rv | 5’-CCTCAAGGCCAGCTCAGA-3’ |  |
| **IRAK3** | Fw | 5’-GCTGCTTGAAAGTCTCTCTGC-3’ | 26 |
|  | Rv | 5’-AGAGCTCTGCGCTGTTGTG-3’ |  |
| **WTN5A** | Fw | 5’-CCCCCTTATAAATGCAACTGTTC-3’ | 48 |
|  | Rv | 5’-ATTGTACTGCAGGTGTACCTTAAAAC-3’ |  |
| **18S** | Fw | 5’-CTTCCACAGGAGGCCTACAC-3’ | 46 |
|  | Rv | 5’-CGCAAAATATGCTGGAACTTT-3’ |  |
| **Glo1** | Fw | 5’-TGGCTTATGAGGATAAAAATGACA-3’ | **SYBR Green** |
|  | Rv | 5’-CAGCTCAAGTGTAGCTTTTCTGG-3’ |  |
